# Clinician Experiences With Tele-Emergency Care: Qualitative Study

**DOI:** 10.2196/96345

**Published:** 2026-10-05

**Authors:** Jessica Faiz, Caroline Gray, Allison Engstrom, Justine Seidenfeld, Anita A Vashi

**Affiliations:** 1 HSR Center for the Study of Healthcare Innovation, Implementation & Policy, VA Greater Los Angeles Healthcare System, United States Department of Veterans Affairs, 1100 Glendon Ave., Ste 1200, Los Angeles, CA, 90024, United States, 1 2012146494; 2 Department of Emergency Medicine, United States Department of Veterans Affairs, Los Angeles, CA, United States; 3 Department of Emergency Medicine, David Geffen School of Medicine at University of California, , Los Angeles, CA, United States; 4 Center for Innovation to Implementation, VA Palo Alto Health Care System, United States Department of Veterans Affairs, Menlo Park, CA, United States; 5 Department of Emergency Medicine, Stanford University, Stanford, CA, United States; 6 Center of Innovation to Accelerate Discovery and Practice Transformation (ADAPT), Durham VA Health Care System, United States Department of Veterans Affairs, Durham, NC, United States; 7 Department of Emergency Medicine, Duke University, Durham, NC, United States

**Keywords:** Telemedicine, emergency medicine, burnout, wellness, virtual care

## Abstract

**Background:**

Emergency departments (EDs) face persistent challenges related to overcrowding, boarding, ambulatory care access barriers, and workforce strain, contributing to compromised patient care and high rates of physician burnout. Virtual care has emerged as a potential strategy to alleviate pressure on emergency care systems. In 2020, the Veterans Health Administration (VA) launched the national Tele-Emergency Care (TEC) program, in which patients who call a call center can be connected to an emergency medicine clinician by phone or video. Although virtual care may help address ED capacity and clinician burnout, the perspectives of emergency medicine–trained clinicians remain limited.

**Objective:**

The aim of this study is to examine the experiences of emergency medicine clinicians participating in VA’s TEC program.

**Methods:**

As part of a national mixed methods evaluation of TEC, we conducted semistructured interviews with clinicians delivering emergency care through TEC between February 2025 and June 2025. Participants (n=15) were recruited via multistage purposeful sampling from 4 of 18 regional TEC programs that varied in geography, volume, duration, and operational models. Interviews explored experiences of providing care in a virtual environment, including perceived benefits and challenges. We performed a descriptive qualitative analysis.

**Results:**

We interviewed 14 physicians and 1 nurse practitioner with formal emergency medicine training. Interviewees described four primary themes: (1) clinical decision-making in a virtual environment; (2) development of the provider-patient relationship; (3) clinician job satisfaction and professional well-being; and (4) challenges. Participants reported that TEC provided perceived opportunities to avoid ED referrals, more focused patient interactions, and improved job satisfaction related to flexible virtual shifts. Reported challenges included filling primary care gaps and performing care coordination tasks.

**Conclusions:**

TEC represents an emerging model of emergency care delivery that clinicians perceive may expand access, prevent avoidable ED visits, and support clinician well-being while also introducing distinct clinical and operational challenges. Our findings can inform the implementation of similar emergency telehealth services in other health systems.

## Introduction

Emergency departments (EDs) have long faced persistent challenges related to overcrowding, boarding, and workforce strain, often driven by structural factors such as limited access to primary care, high inpatient census, and staffing shortages [1,2]. These pressures adversely affect patient care and have contributed to a high-stress clinical environment. Consequently, emergency medicine reports one of the highest rates of physician burnout [3-5]. As these systemic challenges intensify, there is growing urgency to identify innovative care delivery solutions that can mitigate ED crowding while supporting workplace sustainability [6].

The rapid expansion of virtual care during the COVID-19 pandemic has created interest in telemedicine as a potential solution to address downstream pressures on emergency care. The Veterans Health Administration (VA) has been a national leader in virtual care adoption [7], including the development of novel models within emergency medicine [8]. In 2020, the VA launched the national Tele-Emergency Care (TEC) program, which allows veterans who call a VA clinical call center and are triaged to a nurse-led medical advice line for acute symptoms to connect immediately with an emergency medicine clinician via phone or video, rather than being referred directly to the ED (Figure 1). The clinician then evaluates the caller’s condition, provides treatment recommendations, prescribes medications, orders tests, or arranges follow-up care as needed, including advising patients to present in person to an urgent care or ED if ultimately deemed necessary [9].

**Figure 1.**
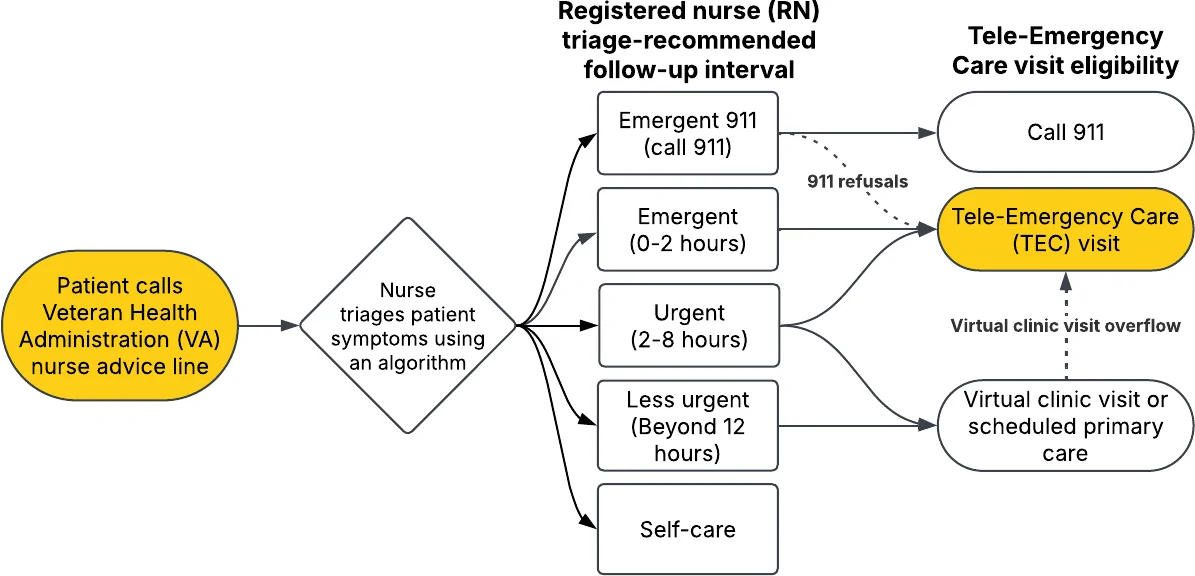
Tele-Emergency Care (TEC) triage workflow from the initial call to the Veterans Health Administration (VA) nurse advice line to the TEC visit.

The VA’s integrated structure, which includes a single national electronic health record spanning 111 EDs, internal ordering and referral pathways, and coordinated follow-up, shapes how TEC operates. However, features of TEC are not exclusive to the VA, as virtual urgent and emergency care services have been implemented at academic medical centers and individual hospitals outside integrated systems [10,11]. VA TEC differs from these models primarily in scale and structure, with unique aspects including national deployment, entry through nurse triage rather than patient self-referral, and clinicians for whom virtual shifts may be part of, rather than additional to, their clinical shift load.

Since 2020, the TEC program has expanded from serving a small number of states to a nationwide program, now employing more than 400 physicians. TEC positions emergency clinicians upstream of the physical ED, engaging patients directly in their home environment with the goal of resolving acute concerns and safely avoiding unnecessary ED visits when possible. Early evaluations of TEC have largely focused on its effects on care use and safety. Prior work suggests that TEC clinicians can resolve veterans’ acute concerns in over half of encounters [9], and another study found that the service was associated with a 5.5% decline in subsequent ED visits without corresponding changes in hospital admission rates or short-term mortality [12].

In addition to its potential impact on ED patient flow, TEC represents a novel workforce model. TEC differs from most telemedicine in both the clinical task and the clinician performing it. Existing telemedicine literature largely describes scheduled encounters with established patients for chronic disease management, behavioral health, or specialty consultation [13-19]. Much like in-person emergency care, TEC encounters are unscheduled, involve undifferentiated acute symptoms, and occur with patients with whom the clinician has no prior relationship, though with full access to the electronic health record. The primary task is acute management for potentially serious or acute conditions. Without a physical examination or immediate test results, clinicians determine whether immediate in-person evaluation would meaningfully change management and, when it would not, determine how best to treat, prescribe, or arrange follow-up. This distinguishes TEC from most other models of telemedicine. The clinician role is likewise atypical. Emergency medicine training centers on the in-person ED environment, while TEC clinicians practice within varying staffing models that range from predominantly virtual roles to models combining TEC with regular in-person ED practice. Prior work on virtual emergency and urgent care reflects different models, including direct-to-consumer visits substituting for urgent care appointments, delivered largely by primary care–trained clinicians [11], and a single-site virtual ED during the COVID-19 pandemic in which some physicians reported that their emergency medicine skills were underutilized [10].

While there is a growing body of literature examining clinician experiences with telemedicine in other clinical settings [13-19], less is known about how emergency medicine clinicians experience delivering virtual care, particularly in virtual emergency care models like TEC. Important unexplored questions include how this care model affects the provider-patient relationship, clinical decision-making, professional identity, and perceptions of burnout.

Understanding the experiences of emergency medicine–trained clinicians is central to evaluating whether TEC can be sustained and scaled. TEC relies on a workforce already facing shortages and high rates of burnout, making clinician willingness to provide virtual care a critical determinant of program capacity. Clinicians are also central to how TEC may influence downstream use [12], as their judgments about risk without a physical examination help determine whether patients can remain at home or require escalation to in-person care.

Thus, the objective of this qualitative study was to examine the experiences of emergency medicine clinicians participating in the VA’s TEC program and to explore how TEC influences clinical decision-making, provider-patient relationships, job satisfaction, and perceived challenges relative to traditional in-person emergency care.

## Methods

### Study Design

As part of a national mixed methods evaluation examining the implementation and impact of VA’s TEC program, we conducted in-depth interviews with clinicians from TEC programs. The qualitative component was designed to elicit clinicians’ experiences delivering emergency care through TEC, identify perceived benefits and challenges of the model, and inform program improvement. We employed a qualitative descriptive approach [20] to analyze interview transcripts, using constant comparison [21] to iteratively organize, refine, and compare and contrast data across transcripts.

We performed a descriptive qualitative analysis and used summation as our analytic goal. We used summation as our analytic goal because this applied evaluation sought a descriptive synthesis rather than theoretical development. The 15 interviews across 4 programs produced detailed accounts across all major interview domains and sufficient recurrence and variation to support the 4 descriptive themes reported. We therefore judged the sample adequate for these bounded aims, although not sufficient to establish national representation, compare static and distributed staffing models, or capture perspectives across all 18 programs. While researchers have traditionally relied on saturation as an analytic benchmark, many are increasingly finding it to be ill-suited for more applied, health services–oriented research [21]. Summation-driven studies are more modest in scope than saturation-based studies that aim for conceptual and theoretical development [21]. Reporting of this qualitative study followed the COREQ (Consolidated Criteria for Reporting Qualitative Research) guidelines, with the completed checklist included in Checklist 1.

### Study Setting and Population

Interviews were conducted between February 2025 and June 2025. We employed a multistage purposeful sampling strategy [22]. In the first stage, we purposefully selected 4 of 18 TEC programs to achieve maximum variation across TEC program type, tenure, geography, and veteran population characteristics (Table 1). In the second stage, the National Emergency Medicine Office asked leads at each of the 4 selected programs to provide the evaluation team with a complete contact list of all TEC staff, rather than to nominate specific clinicians or apply selection criteria. The evaluation team then contacted providers directly via email and Microsoft Teams messages to invite participation in one-on-one interviews. Because leads served as intermediaries in identifying potential participants, the completeness of these lists could not be verified. Of the 16 clinicians contacted, 15 agreed to voluntarily participate in an interview.

**Table 1.** Participants, their training backgrounds, and associated Tele-Emergency Care (TEC) program characteristics.

Characteristics	Values, n (%)
Role	
Physician	14 (93.3)
Doctor of Medicine	11 (78.6)
Doctor of Osteopathic Medicine	3 (21.4)
Nurse practitioner	1 (6.7)
Years in TEC practice (y)	
<2	2 (13.3)
≥2	6 (40)
Not reported	7 (46.7)
Practice region	
Northeast	4 (26.7)
Midwest	3 (20)
West	8 (53.3)
Program serves higher-than-average population of rural veterans	3 (20)
Program volume	
High (≥2 veterans per hour)	6 (40)
Low (<2 veterans per hour)	9 (60)
Program type	
Static: clinician primarily provides TEC services with little to no in-person emergency department shifts	9 (60)
Distributed: clinician primarily provides in-person emergency department care with occasional TEC shifts	6 (40)

### Data Collection and Analysis

The interview guide was developed by our interdisciplinary evaluation team (AE, JF, CG, JS, and AAV), which included qualitative researchers and emergency medicine physicians, 2 of whom worked clinical TEC shifts, with additional input from VA operational partners (Multimedia Appendix 1). The draft guide was then pilot-tested with an emergency medicine physician who also served as a TEC clinician but was not part of the study sample. The guide was largely retained following the pilot, with minor wording clarifications. Interviews explored clinicians’ experiences delivering TEC, challenges with virtual emergency care, needed improvements to the program, and tools and training needs specific to clinicians. Clinicians also discussed the impact of TEC on their overall work satisfaction and its role in addressing burnout. Two trained female qualitative researchers, with backgrounds in sociology (CG) and social work (AE), conducted all interviews. The research team did not have a prior clinical or supervisory relationship with participants. Participant characteristics were recorded from information volunteered during interviews rather than collected through a standardized form; denominators, therefore, vary by characteristic and are reported in Table 1.

Semistructured interviews were conducted using Microsoft Teams, a secure videoconferencing platform. With participant consent, interviews were audio-recorded and professionally transcribed. Interviewers drafted summary memos following each interview to support generative analysis and facilitate debriefings with the broader evaluation team. These debriefings informed iterative refinements to the interview guide, including the addition of follow-up questions or new probes. This approach is consistent with data-driven forms of qualitative inquiry.

Interview transcripts were analyzed using a qualitative descriptive approach that employed a constant comparative analytic technique to further reduce and synthesize data [23,24]. Though typically associated with grounded theory approaches, constant comparison is a way to continually compare previously analyzed data with new data to look for patterns as well as areas of divergence. Our coding process was abductive, involving both “deductive” codes derived from our interview guide and knowledge about the field, as well as “inductive” codes and categories that were based on immersion in the interview data itself [25]. The codebook was developed collaboratively by the evaluation team, informed by the interview guide’s domains and iterative review of early transcripts. Transcripts were uploaded into ATLAS.ti and coded by a single trained qualitative analyst (CG) using the collaboratively developed codebook. Independent double-coding and interrater reliability statistics were not used [25]. The single-coder approach was selected because of evaluation time constraints and limited opportunities to assess coding consistency. To strengthen analytic rigor, coding decisions and emerging categories were discussed with our interdisciplinary evaluation team during twice-monthly team debriefings, providing an additional layer of collaborative review. Regarding positionality, the primary coder (CG) is a social scientist with more than 10 years of experience conducting VA health services research and nearly 10 years evaluating virtual care. AE has more than 10 years of experience in mixed methods research in acute care, including VA program evaluation, as well as prior clinical experience as a social worker in primary care and trauma center settings. CG and AE do not provide TEC. Two emergency medicine physician members of the analytic team (JF and JS) provide TEC, bringing direct clinical and operational knowledge of the program. All emergency medicine physician team members (JF, JS, and AAV) provide in-person clinical care to veterans in VA EDs. Thus, the analysis combined non-TEC analyst perspectives with insider clinical perspectives.

Following coding, team members (AE, JF, CG, JS, and AAV) reviewed and grouped codes into broader categories and identified higher-level themes based on patterns of repetition and emphasis. Finally, quotes were selected to illustrate each of the major themes and subthemes presented in the *Results* section.

### Ethical Considerations

This quality improvement project was reviewed and designated as nonresearch by the Institutional Review Board of Stanford University. The institutional review board has determined that this project does not meet the definition of research as defined in 45 Code of Federal Regulations (CFR) 46.102(d) or the definition of a clinical investigation as defined in 21 CFR 50.3(c). Despite being categorized as nonresearch, oral informed consent was obtained from all participants prior to their involvement in the study. Permission was also granted to audio-record the interviews. All audio recordings were transcribed, and the transcripts were deidentified to ensure the anonymity of participants. This deidentification step was taken to protect the privacy and confidentiality of the participants.

Additionally, to ensure confidentiality, recruitment and interviews were conducted by the evaluation team, and individual responses were not shared with program leadership. Interview participants were provided with a description of the evaluation’s aims, assured that confidentiality would be maintained, and informed that findings would be reported at an aggregate level rather than identified by location or any other potentially identifiable information. Participants did not receive compensation, as VA staff and providers are not allowed to accept compensation for participation in research or evaluation activities.

## Results

### Study Sample and Overview of Themes

Participants included 14 emergency medicine physicians and 1 nurse practitioner, all of whom had formal emergency medicine training and experience (Table 1). The mean interview length was 32 (SD 14) minutes.

Participants described four primary ways that TEC affects the delivery and experience of providing emergency care: (1) clinical decision-making in a virtual environment; (2) development of the provider-veteran relationship; (3) clinician job satisfaction and professional well-being; and (4) challenges of TEC (Table 2).

**Table 2.** Domains and subthemes regarding the Tele-Emergency Care (TEC) clinician experience.

Domain	Subtheme
Clinical decision-making in a virtual environment	Safely avoiding emergency department visits Engaging in shared decision-making Adapting clinical reasoning in the absence of a physical exam
Development of the provider-veteran relationship	Delivering more patient-centered care Creating space to acknowledge veteran-specific perspectives on emergency care
Clinician job satisfaction and professional well-being	Having more one-on-one time with patients Experiencing professional fulfillment through veteran gratitude Improving work-life integration through virtual practice
Challenges of TEC	Filling gaps of primary care Assuming care coordination responsibilities

### Clinical Decision-Making in a Virtual Environment

Emergency medicine clinicians are trained to evaluate, stabilize, and resuscitate patients in a resource-rich environment. In contrast, TEC clinicians described a key component of their jobs as actively working to safely avoid sending veterans to the ED whenever possible and found it rewarding when they could accomplish this. Clinicians reported that they found it rewarding when they could resolve concerns virtually and help veterans avoid an ED visit. This is especially valuable for rural veterans, as highlighted in the following quote:

*…There’s a lot of rural veterans [here] and wide-open spaces. And so if they call in with low blood pressure it could be a three-hour ambulance ride to a hospital to go, oh, yeah, it looks like you took an extra pill, go home. And so by having this program, we can often see them at home and keep them at home.*

[Participant 1]

In traditional ED settings, there are abundant diagnostic resources available, including laboratory testing and imaging, making it easy for clinicians to order these workups even if not strictly necessary. TEC clinicians described their work as requiring more thoughtful consideration of whether in-person evaluation would meaningfully change management. With ready access to the veterans’ electronic health record, clinicians described initially risk-stratifying patients and then communicating their professional recommendations virtually. In addition, though always important, clinicians perceived that shared decision-making was particularly critical for TEC, especially for veterans who strongly wished to avoid going to the ED or those who sought guidance on whether in-person ED evaluation was necessary. Clinicians described collaboratively offering outpatient alternatives to emergency care to honor patients’ care preferences, like in this clinician’s patient care example, which states:

*I agree with you even though your headache is pretty bad. It sounds like it’s been there for a few days. We could order a CAT [computed tomography] scan and you can get that done tomorrow and that kind of thing. And so it avoids ER [emergency room] visits for them that they don’t want to do. ‘Cause often they’re just like, I don’t want to go to the hospital. I just wanted some advice.*

[Participant 1]

When delivering emergency care virtually, participants described how they adapted their medical evaluation in the absence of a traditional physical exam. To compensate for this absence, video visits rather than phone visits were viewed as critical for making informed decisions. Clinicians perceived that visually observing patients in their home environment was essential for assessing functional status and providing a fuller picture of contextual factors that might be impacting the patient’s health, including mobility, baseline independence, access to caregivers, and the safety of the home setting. Clinicians noted that these observations informed risk stratification and helped them determine whether patients could be safely managed at home or required in-person evaluation. As one provider explained:

*I would say video is the gold standard...even if there is nothing—quote, unquote—to see. Like, there’s not a rash or a swollen elbow, or something like that. Because video offers you the opportunity to see the patient in their context, to see their functional status—[to see] them walking around, all of that ties into the history, and the exam, and so it does greatly improve both history-taking and the exam.*

[Participant 5]

Beyond visual assessment, clinicians reported that TEC required greater reliance on patients’ self-knowledge of symptoms and lived experience of chronic conditions. In the absence of vital signs and other objective data typically available during in-person ED visits, providers described engaging more deeply in dialogue to distinguish benign from high-risk presentations. This approach often involved validating patients’ interpretations of their symptoms while applying clinical judgment to determine the need for escalation. As illustrated below, clinicians contrasted these nuanced clinical assessments with protocol-driven triage processes that may default to unnecessary ED referral:

*…people have common sense. I know this pain is because I got hit with a softball over here not ‘cause I’m having a heart attack. And [the triage nurse] is making me go to the ER, I don’t wanna go. So the doc—we can kind of, not always, but we can kind of tease those things out and have a little more judgment than the nurse who’s kind of mandated to somewhat follow a protocol. So I think the veterans are pretty happy when we talk to ‘em and go, hey, look, I agree with you. I think we can send you a muscle relaxer. I don’t think you need to go in and have an x-ray and all that. And then they’re like, oh good, ‘cause it’s 45 minutes away.*

[Participant 1]

### Development of the Provider-Veteran Relationship

In contrast to the fast-paced nature of the in-person ED, where clinicians multitask, have frequent interruptions, and simultaneously manage multiple high-acuity patients, interviewees described the opportunity to have focused, one-on-one interactions with patients on TEC. Clinicians perceived that this uninterrupted time supported rapport-building, facilitated trust, and enabled more patient-centered conversations than are typically possible in the in-person ED environment. One clinician noted:

*…over the past few years there’s been…a disconnect between patients and the health care system…[in TEC] we have patients that are essentially, whether they realize it or not, they’re inviting a doctor into their home…it’s sort of a good old fashioned doctor visit all over again. And at this point in the program, we’re not super time rushed. We’re not being told, oh, you got to see, four patients an hour or things like that. And so you really can get a good sense of how the veteran lives and talk to them about their problem and all.*

[Participant 1]

Clinicians described how TEC created space to acknowledge veteran-specific experiences and values, particularly around ED use. They reported that many veterans preferred to avoid the ED when possible, and that when they could safely honor this preference through virtual care, it reinforced trust and rapport. Clinicians perceived that veterans who experience posttraumatic stress disorder may find the ED environment stressful, with TEC providing a further benefit to such patients. They also noted that some veterans wish to avoid using traditional ED services because they felt that others who might have more urgent needs should be prioritized, and TEC provided those veterans with an additional option for care. One clinician explained:

*And frequently, they’re happy, because you either kept them out of the ER, or what they wanted to know in the first place was if they were overdoing the ER, because veterans are a little different than the community. They actually don’t want to go to the ER if they think that it’s going be wasting somebody’s time or—you know, putting somebody else out., which is way different than what I ran into in the community for 20 years.*

[Participant 2]

### Clinician Job Satisfaction and Professional Well-Being

Clinicians characterized the positive aspects of providing TEC as personally rewarding in terms of minimizing burnout and providing professional meaning. Additionally, some perceived that having time to assist veterans in navigating a complex health care system was particularly rewarding, with one clinician stating:

*I’m able to help people get what they need when they need it, and help them navigate the system because the healthcare system is a complete dumpster fire and disaster…it’s probably the first job in a while where I felt like I was actually able to help people understand and actually get satisfaction from it. Whereas in the ER it’s like, yeah, you save people’s lives, but then outside of that, usually you’re getting yelled at and people are running around screaming and there’s less job satisfaction in that sense. And so I actually really, really enjoy it and I have liked it a lot more than I thought.*

[Participant 8]

Clinicians described veterans’ expressions of gratitude during TEC encounters as a source of professional fulfillment and a further buffer against burnout. They reported that patients expressed appreciation for their TEC visits more often than in the high-stress atmosphere of the ED, and feeling valued reinforced clinicians’ sense of purpose. As one provider explained:

*I really love taking care of our veterans because they’re a great population to serve and they’re very grateful and they’ve seen adversity in their life and I’m really grateful to take care of them. So it’s really helped me with my burnout. It’s really made me a lot more grateful. I really love this job…I’m trying to push the boundaries of virtual care in terms of getting patients on the line talking about their symptoms…*

[Participant 9]

Clinicians also frequently described TEC as a positive change of pace that offered greater schedule flexibility and improved work-life integration. Among clinicians who continued to practice in-person emergency medicine, some described benefits of combining virtual and in-person work, including the ability to maintain procedural skills while periodically working from home. Participants also highlighted benefits of virtual work more generally, including reduced late-night commuting, more time with family, decreased exposure to illness, and fewer in-person distractions. However, these favorable experiences were not universal. Clinicians also described frustration when TEC encounters centered more on administrative tasks, care coordination, or concerns outside the traditional scope of emergency medicine.

### Challenges of TEC

Despite the reported benefits of TEC, clinicians also described less favorable aspects of the virtual care model, and perspectives differed regarding some responsibilities. Clinicians noted that TEC encounters frequently involved clinical issues outside the traditional scope of emergency medicine, particularly the evaluation and management of chronic or subacute conditions typically addressed in primary care. Some clinicians valued the opportunity to fill these gaps in primary care access, whereas others viewed these responsibilities as a less optimal use of emergency medicine expertise. For example, a clinician explained:

*And, you know, nobody gets into primary care quickly at the VA, typically, so it’s kind of nice that we can have these workup pieces in place prior to their appointment, otherwise it pushes out getting an answer for months, and if somebody’s not feeling well or missing work because they can’t walk on their leg, at least we are moving the ball in the right direction much quicker. I do feel like this program fills in the gap for primary care quite a bit. Not that we are functioning as primary care, but we’re kind of moving things along while we’re trying to coordinate that.*

[Participant 6]

Clinicians described how both the VA system and the broader health care system are still challenging to navigate within TEC. While some clinicians viewed helping patients navigate a fragmented system as an opportunity to help move care forward, others expressed concern that these responsibilities extended beyond the intended role of emergency medicine–trained clinicians. They perceived that TEC served not only as a means of addressing acute clinical needs but also as a way to help veterans navigate a complex, fragmented system, suggesting that additional supports for veterans may be needed to address these challenges. A clinician explained:

*The system is very daunting to navigate on their own. And the resources are scarce in these smaller, rural communities. So sometimes it takes a little thinking outside of the box, being able to look things up, to help them figure out next steps.*

[Participant 6]

In this example, the clinician reported being able to assist the veteran, but time constraints and other obstacles may prevent some TEC clinicians from offering this additional help. Finally, in some cases, clinicians reported that patients had already experienced system inefficiencies, such as multiple phone transfers or an inability to reach their outpatient clinicians, before reaching TEC. These inefficiencies at times contributed to patient frustration and made TEC encounters feel suboptimal. One interviewee noted:

*And so there can be periods of time where the patient is waiting. On those days, I’ve had patients be very upset that they’ve been transferred from person-to-person-to-person and then by the time they’ve gotten to me they’ve already been on the phone for an hour and a half. And they’re frustrated. And I don’t fully know how to fix that, only because I don’t fully understand that very front-end process, but again, we’re trying to figure out what we can do on our end to minimize patients waiting.*

[Participant 6]

These experiences suggest that TEC may shift, rather than eliminate, some of the burdens encountered in traditional in-person emergency care, particularly when clinicians are asked to compensate for gaps elsewhere in the health care system.

## Discussion

### Overview and Interpretation of Findings

Our study provides insight into emergency medicine clinicians’ experiences delivering care through VA’s novel TEC program, which positions emergency clinicians upstream of the ED to address urgent concerns in veterans’ home environments. Compared with in-person emergency care, clinicians described how TEC reshapes clinical decision-making, the provider-patient relationship, and clinician well-being. Although many participants perceived benefits, including greater schedule flexibility and more focused patient interactions, others described challenges related to care coordination, work outside the traditional scope of emergency medicine, and system inefficiencies.

Prior VA-based quantitative studies have demonstrated that TEC can resolve acute concerns without ED referral in a substantial proportion of encounters and reduce subsequent ED use without adverse effects on safety outcomes [9,12]. An important feature of TEC is its use of emergency medicine clinicians for virtual evaluation and disposition decisions. Research in other integrated health systems similarly suggests that this upstream, physician-involved virtual approach can safely reduce reliance on in-person emergency care. A Kaiser Permanente study found that physician-directed telephone triage for patients with chest pain was associated with fewer ED referrals and shorter calls than nurse-directed triage, without significant differences in hospital admission or short-term mortality [26]. Our qualitative findings add context to how virtual emergency care is delivered in practice to guide these disposition decisions. Clinicians reported the importance of video-based encounters for assessing functional status, home environment, and available support, noting that these contextual insights supported decisions about when virtual management might be appropriate and when in-person evaluation was warranted. From the patient’s perspective, prior literature has shown that patients also prefer video over phone visits when engaging with clinicians, as it facilitates communication and connection, although preferences may vary by visit type [27-29]. For example, brief or straightforward concerns such as discussing test results may be well suited to phone-based encounters.

The challenges clinicians described in TEC are largely consistent with those encountered during in-person emergency care. As the safety net of the health care system, EDs frequently fill gaps created by limited primary care access, high demand for clinicians, and long waits for outpatient appointments. Patients often present to the ED because they struggle to navigate the system, including coordinating specialty care. Patients’ frustration stemming from system inefficiencies (eg, multiple phone transfers or difficulty reaching outpatient clinicians) that clinicians reported also mirrors common in-person ED experiences, where overcrowding, delays, fragmented communication, and lack of timely follow-up are frequent drivers of dissatisfaction. Notably, clinicians differed in how they viewed addressing these responsibilities in TEC. Some found helping veterans navigate fragmented care professionally rewarding, whereas others viewed care coordination and management of nonacute concerns as extending beyond the intended role of emergency medicine–trained clinicians.

Participants described schedule flexibility and focused patient interactions as benefits of TEC. Among clinicians who also maintained in-person ED practice, some valued combining the 2 settings, noting that in-person work could help preserve procedural skills and professional identity while virtual shifts reduced commuting time and occupational exposure to illness. Prior studies in other specialties report mixed clinician experiences with hybrid practice, including comparable job satisfaction and concerns about reimbursement [30-32]. These accounts suggest that mixed virtual-in-person roles may be attractive to some clinicians, but they should not be interpreted as a uniform preference. Sixty percent of participants in our study were affiliated with static programs (where the clinician primarily provides TEC services with little to no in-person ED shifts), and we did not systematically assess staffing preferences or each clinician’s distribution of virtual and in-person work outside TEC. We therefore cannot compare static and distributed programs (where the clinician primarily provides in-person ED care with occasional TEC shifts) or conclude that a hybrid model is superior. Future work should directly evaluate how staffing configuration relates to clinician satisfaction, skill maintenance, retention, and program capacity. Clinicians perceived that more focused, uninterrupted virtual encounters facilitated more patient-centered relationships; however, patient satisfaction and trust were not directly assessed in this study. Additionally, these favorable perceptions should be interpreted in light of potential selection bias, as clinicians who voluntarily participated may have been more engaged with or supportive of TEC.

Across staffing models, clinicians reported that delivering emergency care through TEC required adapting traditional ED clinical reasoning to a virtual setting, with greater reliance on history and physical exam via video, and shared decision-making in the absence of immediate diagnostics. Clinicians perceived that this model may be particularly useful for veterans who face geographic or logistical barriers to timely in-person acute care. Based on these reported experiences, training and onboarding for virtual care positions should explicitly address clinical decision-making without a traditional physical exam and shared decision-making strategies tailored to virtual care. Given how widespread virtual care options across specialties have become since COVID-19, these competencies could be introduced as early as medical school and reinforced in residency curricula [33-35].

Notably, our participants did not describe the concerns most prominent in prior studies of virtual emergency and urgent care. In a single-site virtual ED, a subset of physicians reported that their emergency medicine skills were underutilized and that patient issues were often unresolved [10], and clinicians delivering direct-to-consumer tele-urgent care described tension arising from patient expectations for prescriptions or testing [11]. Neither pattern appeared in our interviews. Structural differences may contribute to this finding. For instance, TEC encounters are initiated through nurse triage rather than patient self-referral, virtual shifts are incorporated into clinicians’ regular schedules rather than added to them, and clinicians are salaried rather than compensated per encounter. These features may also influence how readily virtual emergency care can be implemented outside the VA. Although physician-led virtual urgent and emergency care has been implemented at academic medical centers and individual hospitals [10,11], nonintegrated systems may have more fragmented access to medical records, ordering, referrals, and follow-up. Similar limitations can occur within the VA when veterans receive care outside the system. Reimbursement and referral pathways may be particularly important, as they may influence not only the feasibility of virtual emergency care but also clinician workload, patient expectations, and the clinical encounter itself.

Several implications emerge for the implementation and expansion of virtual emergency care, more generally, given our findings. Further work is needed to define and evaluate quality and safety metrics, particularly for high-acuity presentations. This is especially important, given that TEC clinicians must adapt traditional emergency medicine decision-making to a setting without a hands-on examination or immediate diagnostic testing. Triage processes should also be evaluated to ensure that emergency medicine–trained clinicians are deployed where their expertise adds the greatest value. Future work should evaluate how different staffing configurations and reimbursement models influence clinician experience, maintenance of clinical skills, clinician retention, and program sustainability. Finally, medicolegal considerations, particularly those related to managing higher-acuity presentations in virtual settings, warrant evaluation as these models evolve.

### Limitations

This study has limitations. The veteran patient population is predominantly older, White, and male, which may limit the transferability of findings to more diverse patient populations. The VA is an integrated national health system which may limit the applicability of aspects of the program to nonintegrated health systems. The national scope of the study sample is a strength, and clinicians who agreed to be interviewed may have been more familiar with TEC, leading to more informed insights. However, although program leads were asked to provide complete staff contact lists rather than nominate clinicians, we could not independently verify the completeness of those lists; eligible clinicians may therefore have been omitted before recruitment. Among those contacted, clinicians who volunteered may also have been more engaged with or favorable toward TEC, introducing additional sampling bias.

Furthermore, our sample of TEC providers limits the transferability of our findings. Our sampling strategy did not capture clinicians who opted out of or discontinued participation in TEC and providers who agreed to participate overrepresented some geographic regions and the static operational model. TEC clinicians reported their perceptions of medical decision-making and job satisfaction; however, this study did not directly measure use, patient safety, or clinician retention outcomes. Finally, since only clinicians were interviewed, perceived improvements in patient-centeredness, satisfaction, and trust do not represent the veteran perspective, and future work should elicit patient perspectives.

### Conclusions

Emergency medicine clinicians report that TEC can function to meet veterans where they are, providing evaluation in a virtual setting to prevent avoidable ED visits and addressing access challenges, particularly for rural veterans. For clinicians who combined TEC with in-person emergency practice, this hybrid staffing was perceived as supporting clinician well-being through greater schedule flexibility and more focused time with patients, which participants described as helping them reconnect with their core motivation for practicing medicine. However, this study was not designed to compare staffing models or determine whether one configuration was preferable. Our findings can help inform the development and implementation of similar virtual emergency services and staffing models in other settings.

## Supplementary material

10.2196/96345 Multimedia Appendix 1 Tele-Emergency Care clinician interview guide.

10.2196/96345 Checklist 1 COREQ checklist.

## Abbreviations

COREQ: Consolidated Criteria for Reporting Qualitative Research
ED: Emergency Department
TEC: Tele-Emergency Care
