# Supplementary material for: Clinician Experiences With Tele-Emergency Care: Qualitative Study
Source: J Med Internet Res. 2026 Oct 5;28:e96345. doi: 10.2196/96345 (PMC13637982; doi:10.2196/96345)
Supplement: Supplementary file 1 [file jmir-v28-e96345-s001.docx]

**Supplement 2.** Tele-Emergency Care Clinician Interview Guide

Interviewer: *Thank you for taking the time to meet with me. This interview will last approximately 30-60 minutes. If you have limited time, we will structure the interview session to fit your work schedule.* *Anonymity will be protected in that your identity will be kept confidential by the research team and identifiers will be kept separate from the coded data. Results will be reported in aggregate and will therefore be anonymous in such reports. Your participation is voluntary – you have the right not to answer questions and to stop the interview at any time. Everything you say will be kept confidential – we will not share anything you say directly with anyone else than our project team. Before I begin, do you have any questions? Do I have your consent to record this interview?*

1. Briefly **describe your role** with the Tele-EC program (e.g., FTE, level of involvement in Tele-EC/other settings)
   a. How long have you been a Tele-EC provider?
   b. How many shifts do you do per month?
   c. What **other clinical services do you provide** (if any)? **(e.g., in-person ED, VCV)**
2. What has your experience been like with the **handoff process from the Tele-EC RNs OR the CCC RNs?**
   a. What has been working well or not well?
3. What are some **obstacles to creating virtual clinical experience** that’s comparable to an in-person visit? (Probes: privacy, technology, and other technical considerations)
4. What types of patient **populations** or **chief complaints** have you found **most difficult** to **manage virtually** and why?
   a. Which have been the **most successful**?
5. What **factors** influence your decision to **choose a phone encounter versus a video encounter**?
   a. Is **patient preference the primary driver**, or do other considerations play a role (e.g., clinical condition, technology access)?
   b. In your experience, are there certain clinical scenarios where you **believe one modality (phone or video)** is **more appropriate** than the other? Why?
6. Do you feel that your **ability to assess** the patient is **impacted** by the type of encounter (**phone vs. video**)? How so?
   a. Do you find that you’re **more likely to schedule follow-up appointments** or request additional information (e.g., labs, imaging) depending on whether the encounter was phone or video?
   b. Have you noticed any difference in **clinical outcomes** depending on whether the encounter is done by phone or video?
7. How do you handle situations where you feel **an in-person evaluation is necessary**?
   a. If it is emergent care (meaning they need to be seen at an ED or urgent care), how do you **make suggestions regarding VA vs community sites**?
   b. **If it is not immediate** (that is they can be seen by their PCP), how do you handle that?
8. Do you think you have been able to **help Veterans avoid an unnecessary ED visit**? **How** or **why**?
9. How **often** do you **place orders for Tele-EC**? What **type of orders** do you most often order? (Probes: labs, imaging, specialty care consultation)
   a. What **factors** do you consider when **deciding to place orders**? Do **patient characteristics** affect which **orders you place?** (Probes: age, disability, location, access to care, phone vs. video visit)
10. What is the **follow-up process** for these orders (e.g., who follows up on these orders)? What has been your **experience with prescribing**?
11. How do you **determine** whether to mark an encounter as **“resolved?”** When you mark **“Issue Resolved”** at the end of the visit, what leads you to do so? (Probes: patient did not require follow-up, orders placed, medication prescribed)
12. What **tools or resources** would help you provide **better virtual emergency care**?
13. What **suggestions** do you have **for improving** the Tele-EC program?
    a. What **additional resources** would be helpful for you as a Tele-EC provider (e.g., addition of a service [GS13 pharmacist, social worker], technology upgrades, e-prescribing)
    b. If program has a social worker: **how do you utilize Tele-EC social work services**?
    c. If program has Tele-EC RN: **how do you utilize Tele-EC RN services**?
14. How has providing Tele-EC impacted your **job satisfaction and workload**?
15. What **support** do you get **from your hospital and/or VISN and/or Tele-EC leadership** as a Tele-EC provider?
    a. Probes: responsiveness to concerns
