# Supplementary material for: Clinician Experiences With Tele-Emergency Care: Qualitative Study
Source: J Med Internet Res. 2026 Oct 5;28:e96345. doi: 10.2196/96345 (PMC13637982; doi:10.2196/96345)
Supplement: Supplementary file 2 [file jmir-v28-e96345-s002.docx]

# Supplement 1. COREQ Checklist

| Item No. | Checklist Item | Page No. |
| --- | --- | --- |
| Domain 1: Research Team and Reflexivity |  |  |
| Personal Characteristics |  |  |
| 1 | Interviewer/facilitator: Which author/s conducted the interview or focus group? | 8 |
| 2 | Credentials: What were the researcher’s credentials? (e.g., PhD, MD) | Title page |
| 3 | Occupation: What was their occupation at the time of the study? | 8 |
| 4 | Gender: Was the researcher male or female? | 8 |
| 5 | Experience and training: What experience or training did the researcher have? | 8,9 |
| Relationship with Participants |  |  |
| 6 | Relationship established: Was a relationship established prior to study commencement? | 8 |
| 7 | Participant knowledge of the interviewer: What did participants know about the researcher? (e.g., personal goals, reasons for doing the research) | 8 |
| 8 | Interviewer characteristics: What characteristics were reported about the interviewer/facilitator? (e.g., bias, assumptions, reasons and interests in the research topic) | 8 |
| Domain 2: Study Design |  |  |
| Theoretical Framework |  |  |
| 9 | Methodological orientation and theory: What methodological orientation was stated? (e.g., grounded theory, discourse analysis, ethnography, phenomenology, content analysis) | 6-8 |
| Participant Selection |  |  |
| 10 | Sampling: How were participants selected? (e.g., purposive, convenience, consecutive, snowball) | 7 |
| 11 | Method of approach: How were participants approached? (e.g., face-to-face, telephone, mail, email) | 7 |
| 12 | Sample size: How many participants were in the study? | 7 |
| 13 | Non-participation: How many people refused to participate or dropped out? Reasons? | 7 |
| Setting |  |  |
| 14 | Setting of data collection: Where was the data collected? (e.g., home, clinic, workplace) | 7 |
| 15 | Presence of non-participants: Was anyone else present besides the participants and researchers? | N/A |
| 16 | Description of sample: What are the important characteristics of the sample? (e.g., demographic data, date) | 11 (Table 1) |
| Data Collection |  |  |
| 17 | Interview guide: Were questions, prompts, guides provided? Was it pilot tested? | 7 |
| 18 | Repeat interviews: Were repeat interviews carried out? If yes, how many? | N/A |
| 19 | Audio/visual recording: Did the research use audio or visual recording to collect the data? | 8 |
| 20 | Field notes: Were field notes made during and/or after the interview or focus group? | 8 |
| 21 | Duration: What was the duration of the interviews or focus group? | 10 |
| 22 | Data saturation: Was data saturation discussed? | 6-7 |
| 23 | Transcripts returned: Were transcripts returned to participants for comment and/or correction? | N/A |
| Domain 3: Analysis and Findings |  |  |
| Data Analysis |  |  |
| 24 | Number of data coders: How many data coders coded the data? | 9 |
| 25 | Description of the coding tree: Did authors provide a description of the coding tree? | N/A |
| 26 | Derivation of themes: Were themes identified in advance or derived from the data? | 9 |
| 27 | Software: What software, if applicable, was used to manage the data? | 9 |
| 28 | Participant checking: Did participants provide feedback on the findings? | N/A |
| Reporting |  |  |
| 29 | Quotations presented: Were participant quotations presented to illustrate the themes/findings? Was each quotation identified? (e.g., participant number) | 11-19 |
| 30 | Data and findings consistent: Was there consistency between the data presented and the findings? | 11-19 |
| 31 | Clarity of major themes: Were major themes clearly presented in the findings? | 11-19 |
| 32 | Clarity of minor themes: Is there a description of diverse cases or discussion of minor themes? | 11-19 |
